# Youth-developed recommendations on public health planning for future pandemics or public health emergencies: a national Delphi study

**DOI:** 10.17269/s41997-025-01109-2

**Published:** 2025-10-01

**Authors:** Meaghen Quinlan-Davidson, Kristin Cleverley, Skye Barbic, Darren Courtney, Gina Dimitropoulos, Lisa D. Hawke, Nadia Nandlall, Clement Ma, Matthew Prebeg, J. L. Henderson

**Affiliations:** 1 Centre for Addiction and Mental Health, Toronto, Ontario Canada; 2 Dalla Lana School of Public Health, University of Toronto, Toronto, Ontario Canada; 3 Department of Psychiatry, University of Toronto, Toronto, Ontario Canada; 4 Lawrence Bloomberg Faculty of Nursing, University of Toronto, Toronto, Ontario Canada; 5 Faculty of Medicine, University British Columbia, Vancouver, British Columbia Canada; 6 Foundry BC, Vancouver, British Columbia Canada; 7 Faculty of Social Work, University of Calgary, Calgary, Alberta Canada; 8 York University, Toronto, Ontario Canada

**Keywords:** Youth mental health and substance use, Preparedness, Delphi study, Consensus, Santé mentale et consommation de substances chez les jeunes, Préparation, Étude Delphi, Consensus

## Abstract

**Objectives:**

To generate concrete, youth-derived recommendations for government, policymakers, and service planners to support public health planning for the next pandemic or public health emergency.

**Methods:**

Using a virtual, modified Delphi, Youth Delphi Expert Panel Members rated recommendation items over three rounds, with the option to create their own recommendations items. ‘Consensus’ was defined a priori if ≥ 70% of the entire group, or subgroups of youth (e.g., age, race/ethnicity, gender and sexual identities), rated items at a 6 or 7 (on a 7-point Likert scale). Items that did not achieve consensus were dropped. Content analysis was used for qualitative responses in Rounds 1 and 2. Youth were engaged as members of an expert advisory committee throughout the design, implementation, and interpretation of findings.

**Results:**

A total of *n* = 40 youth participated in Round 1 with good retention (> 95%) in subsequent rounds. Youth endorsed eleven recommendations to support public health planning for future pandemics or public health emergencies. Youth prioritized easily accessible and understandable information about pandemics; equitably and efficiently distributed vaccines; increased awareness of timely and accessible mental health and substance use services in schools, workplaces, and communities; and greater investment in free or inexpensive MHSU services.

**Conclusions:**

For Canada to move forward in a relevant, efficient, and ethically sound manner, decisions must be guided by the population that these decisions affect. These recommendations can be used to guide Canada’s strategies and policies to prepare for future public health emergencies and pandemics, prioritizing the needs of youth, families/caregivers, and communities.

## Résumé

**Objectifs:**

Générer des recommandations concrètes, inspirées des jeunes, à l’intention du gouvernement, des décideurs politiques et des planificateurs de services afin de soutenir la planification de la santé publique pour la prochaine pandémie ou urgence de santé publique.

**Méthodes:**

À l'aide d'une méthode Delphi virtuelle modifiée, les membres du panel d'experts de la méthode Delphi Jeunesse ont évalué les éléments de recommandation sur trois phases, avec la possibilité de créer leurs propres éléments. Un « consensus» a été défini a priori si ≥ 70% de l'ensemble du groupe, ou de sous-groupes de jeunes (par exemple, âge, origine ethnique, genre et identité sexuelle), ont attribué aux éléments une note de 6 ou 7 (sur une échelle de Likert à 7 points). Les éléments n'ayant pas fait l'objet d'un consensus ont été abandonnés. Une analyse de contenu a été utilisée pour les réponses qualitatives lors des phases 1 et 2. Les jeunes ont été impliqués en tant que membres d'un comité consultatif d'experts tout au long de la conception, de la mise en oeuvre et de l'interprétation des résultats.

**Résultats:**

Au total, n = 40 jeunes ont participé au premier tour, avec un bon taux de rétention (> 95%) lors des tours suivants. Les jeunes ont approuvé onze recommandations visant à soutenir la planification de la santé publique en cas de futures pandémies ou urgences de santé publique. Ils ont privilégié une information facilement accessible et compréhensible sur les pandémies; une distribution équitable et efficace des vaccins; une meilleure sensibilisation à l'accessibilité et à la rapidité des services de santé mentale et de toxicomanie dans les écoles, les lieux de travail et les communautés; et un investissement accru dans les services MHSU gratuits ou peu coûteux.

**Conclusions:**

Pour que le Canada progresse de manière pertinente, efficace et éthique, les décisions doivent être guidées par la population qu’elles touchent. Ces recommandations peuvent servir à orienter les stratégies et les politiques du Canada pour se préparer aux futures urgences de santé publique et pandémies, en accordant la priorité aux besoins des jeunes, des familles, des aidants et des collectivités.

## Introduction

The spread of the coronavirus disease (COVID-19) has been associated with substantial mental health and substance use (MHSU) concerns globally (Chacon et al., 2021). Notwithstanding the impact that the COVID-19 pandemic has had on populations around the world, the burden and risks associated with MHSU concerns have not been equally experienced, with youth (12–25 years) among those disproportionately affected (Chacon et al., 2021).

In Canada, multiple studies showed an increase in youth-reported symptoms, including anxiety, depression, loneliness, helplessness, and substance use over the pandemic period (Hawke et al., 2021a, 2021b; Quinlan-Davidson et al., 2023). Variations in MHSU outcomes were reported among subgroups of youth over the pandemic period. Previous longitudinal cohort research showed that younger youth (14–17 years), youth who identify as Trans, nonbinary, or gender diverse, and those living in urban areas had greater mental health concerns compared to their counterparts (Hawke et al., 2020; Quinlan-Davidson et al., 2023). At the same time, the exacerbation of MHSU concerns among youth was associated with service disruptions (Hawke et al., 2021a, 2021b). The increased MHSU burden, coupled with gaps in the provision of MHSU services, has highlighted lessons learned for the country on planning, response, and adaptation for future public health emergencies and pandemics (Serchen et al., 2023).

Despite the negative MHSU impacts of the COVID-19 pandemic, there is also evidence showing positive effects for some youth. From a strengths-based perspective, a systematic review (Naff et al., 2022) showed that positive coping strategies and resilience were associated with decreased MHSU problems by enhancing youth’s sense of control over the pandemic (Zhang et al., 2020). Positive coping strategies associated with improved mental health included problem-focused coping, humour, exercising, maintaining a routine, friend and family support, and focusing on schoolwork (Cauberghe et al., 2021; Dewa et al., 2021; Duan et al., 2020; Naff et al., 2022). Also, youth were able to create change and support their community through online activism and in-person activities. For example, youth helped coordinate and participate in social and political movements, like the Black Lives Matter (BLM) movement and election campaigning (Schoon et al., 2025; Wilf et al., 2023). Youth also participated in volunteering and community service by delivering food, medication, and hygiene products to equity-deserving communities (Schoon et al., 2025).

As public health restrictions have been reduced across the country, it is important to consider public health planning for future pandemics and emergencies. Given that the COVID-19 pandemic has demonstrated itself to be a contributing factor to poor MHSU among youth (Quinlan-Davidson et al., 2023), it is imperative to focus on public health planning recommendations to optimize Canada’s future response (Thomas et al., 2021). Understanding youth MHSU needs and concerns requires identifying how youth recommend preparing for future pandemics to develop tailored and relevant interventions and services that address their needs (Morganstein, 2022). These interventions and strategies are particularly important for subgroups of youth who experience poorer service access and quality. Indeed, a scoping review has shown that Black youth experience longer wait times, complex geographic and financial barriers, and Anti-Black Racism in MHSU services (Fante-Coleman & Jackson-Best, 2020). Similarly, Indigenous youth are significantly impacted by health inequities, poor service access, unwelcoming environments, and culturally inappropriate care (Blanchet-Cohen et al., 2011). Further, transgender and gender diverse (TGD) youth have reported challenges to accessing quality MSHU services (Roberts & Fantz, 2014) and lower satisfaction with treatment compared to their cisgender counterparts (Kidd et al., 2022). Reducing and rectifying inequities in service access is important because these inequities are unjust, discriminatory, and preventable. When unaddressed, these inequities lead to a greater burden of disease, shorter life expectancy, poorer health outcomes and quality of life, and poverty (Gómez et al., 2021; WHO, 2025). Importantly identifying public health strategies now will help reduce future health risks (Thomas et al., 2021).

At the Margaret and Wallace McCain Centre for Child, Youth and Family Mental Health within the Centre for Addiction and Mental Health (CAMH) in Toronto, Canada, youth from the Youth Engagement Initiative (YEI) would like to support policy- and decision-makers to respond more effectively to the next pandemic or public health emergency. The YEI team wants to use approaches that support and protect youth MHSU and wellness, building on the successes achieved during the COVID-19 pandemic while examining lessons learned. Amplifying youth voices in the creation of recommendations for system and service planning helps improve access, enhances engagement, better tailors the care to youth needs, and increases satisfaction with health services (Henderson. J.L. et al., 2018). Our team conducted a Delphi study on youth-generated recommendations for Canada’s post-pandemic recovery from COVID-19 (Quinlan-Davidson et al., 2025). Findings showed that youth prioritized post-pandemic strategies that focused on (i) implementing effective, accessible, and low-cost MHSU services within schools, workplaces, and communities; (ii) greater awareness about MHSU services in workplaces and schools; (iii) integrating MHSU education into schools; (iv) prioritizing health and well-being in schools and workplaces; and (v) increased access to jobs that pay a living wage.

As a consensus-building technique, Delphi studies make the priorities of participants with expertise clear, engaging them fully in research (Powell, 2003). While there is some previous literature on recommendations for future pandemics and public health emergencies (Serchen et al., 2023), information on consensus among a diverse group of youth about recommendations that are truly relevant to them is lacking. As such, we conducted a national Delphi study to determine youth’s public health planning priorities and recommendations, based on their COVID-19 pandemic experiences. The objective of the study was to generate concrete, youth-derived recommendations for government, policymakers, and service planners to support public health planning for the next pandemic or public health emergency in youth-appropriate manners to maximize health and well-being while minimizing harms.

## Methods

To develop and rank recommendations, the study applied a virtual, modified Delphi technique (Hsu & Sandford, 2007). This approach is a systematic method to establish consensus-based statements relevant to a topic of interest. In contrast to a focus group approach, the intent is to distribute influence from multiple representative perspectives. The first step involves generating initial candidate consensus statements from prior research (see below for details specific to this study). The next step involves assembling a panel whose members represent the desired perspectives. Subsequently, the research team presents candidate consensus statements to the panel members to rank on the level of importance over consecutive rounds, where statements showing consensus are carried over to the next round. Panel members have the option to suggest new candidate consensus statements for future rounds. Each panel member’s response is anonymous to other members and is intended to represent their own opinions and reasoning. The result is a list of actionable consensus-based statements. The Guidance on Conducting and Reporting Delphi Studies (CREDES) Checklist was followed (Jünger et al., 2017) (Table 1.). The Delphi studies were administered via REDCap, an online platform (Harris et al., 2009) and approved by CAMH’s Research Ethics Board in Toronto, Canada (144/2021).

**Table 1.** Guidance on Conducting and Reporting Delphi Studies (CREDES) Checklist

		Page
Rational for Delphi technique		
1	*Justification.* The choice of the Delphi technique as a method of systematically collating expert consultation and building consensus needs to be well justified. When selecting the method to answer a particular research question, it is important to keep in mind its constructivist nature	4–5
**Planning and design**		
2	*Planning and process.* The Delphi technique is a flexible method and can be adjusted to the respective research aims and purposes. Any modifications should be justified by a rationale and be applied systematically and rigorously	5
3	*Definition of consensus.* Unless not reasonable due to the explorative nature of the study, an a priori criterion for consensus should be defined. This includes a clear and transparent guide for action on (a) how to proceed with certain items or topics in the next survey round, (b) the required threshold to terminate the Delphi process, and (c) procedures to be followed when consensus is (not) reached after one or more iterations	10
**Study conduct**		
4	*Informational input.* All material provided to the expert panel at the outset of the project and throughout the Delphi process should be carefully reviewed and piloted in advance in order to examine the effect on experts’ judgements and to prevent bias	6
5	*Prevention of bias.* Researchers need to take measures to avoid directly or indirectly influencing the experts’ judgements. If one or more members of the research team have a conflict of interest, entrusting an independent researcher with the main coordination of the Delphi study is advisable	No conflicts reported
6	*Interpretation and processing of results.* Consensus does not necessarily imply the ‘correct’ answer or judgement; (non)consensus and stable disagreement provide informative insights and highlight differences in perspectives concerning the topic in question	10
7	*External validation.* It is recommended to have the final draft of the resulting guidance reviewed and approved by an external board or authority before publication and dissemination	10
**Reporting**		
8	*Purpose and rationale.* The purpose of the study should be clearly defined and demonstrate the appropriateness of the use of the Delphi technique as a method to achieve the research aim. A rationale for the choice of the Delphi technique as the most suitable method needs to be provided	4–5
9	*Expert panel.* Criteria for the selection of experts and transparent information on recruitment of the expert panel, sociodemographic details including information on expertise regarding the topic in question, (non)response and response rates over the ongoing iterations should be reported	6
10	*Description of the methods.* The methods employed need to be comprehensible; this includes information on preparatory steps (How was available evidence on the topic in question synthesised?), piloting of material and survey instruments, design of the survey instrument(s), the number and design of survey rounds, methods of data analysis, processing and synthesis of experts’ responses to inform the subsequent survey round and methodological decisions taken by the research team throughout the process	5–10
11	*Procedure.* Flow chart to illustrate the stages of the Delphi process, including a preparatory phase, the actual ‘Delphi rounds’, interim steps of data processing and analysis, and concluding steps	6–8
12	*Definition and attainment of consensus.* It needs to be comprehensible to the reader how consensus was achieved throughout the process, including strategies to deal with non-consensus	10
13	*Results.* Reporting of results for each round separately is highly advisable in order to make the evolving of consensus over the rounds transparent. This includes figures showing the average group response, changes between rounds, as well as any modifications of the survey instrument such as deletion, addition or modification of survey items based on previous rounds	10–13
14	*Discussion of limitations.* Reporting should include a critical reflection of potential limitations and their impact of the resulting guidance	17
15	*Adequacy of conclusions.* The conclusions should adequately reflect the outcomes of the Delphi study with a view to the scope and applicability of the resulting practice guidance	18
16	*Publication and dissemination.* The resulting guidance should be clearly identifiable from the publication, including recommendations for transfer into practice and implementation	17 + this publication

*Source*: Jünger S, Payne SA, Brine J, Radbruch L, Brearley SG. Guidance on Conducting and REporting DElphi Studies (CREDES) in palliative care: Recommendations based on a methodological systematic review. Palliat Med. 2017 Sep;31(8):684–706. 10.1177/0269216317690685

### Youth expert advisory committee

Following principles outlined by the Canadian Institutes of Health Research Strategy for Patient-Oriented Research (SPOR) (CIHR, 2023) and the McCain Model of Youth Engagement (Darnay et al., 2019), a Youth Expert Advisory Committee was established with support from the Youth Engagement Initiative team. The Committee included two youth engagement specialists (CAMH youth employees who support the implementation of youth engagement activities and facilitate the relationship between research teams and youth advisors) and three youth advisors (youth WHO consult and collaborate on project activities) 12 to 25 years of age, each with lived/living MHSU-related experiences. This Committee met every 3 to 4 months. The Committee advised on study design, starting recommendations, implementation, interpretation, and language of Delphi findings, and knowledge translation products. The Committee members received honoraria for their time ($30 honorarium for their participation).

### Youth Delphi expert panel members

Youth Delphi Expert Panel Members were recruited from two pre-existing CAMH studies in Ontario (Hawke et al., 2020), Canada, and through internal CAMH networks. Youth were eligible to participate if they were: 12–25 years of age; lived in Canada; and had lived/living experience of MHSU concerns at the time of the study. Members of the Expert Advisory Committee were ineligible to participate on the panel. The study aimed to recruit *n* = 40 youth, which is within the recommended Delphi panel size range (Hasson et al., 2000). Youth were provided a $35 honorarium for their participation in each round of the study.

### Survey development

Initial candidate recommendations were derived from qualitative responses from participants in a pre-existing longitudinal, cohort CAMH-based study (Hawke et al., 2021a, 2021b) in August 2021. Youth participants in this study provided open-ended responses to 12 questions on planning for a future pandemic or public health emergency (Table 2). Members of the research team analysed responses to these questions, following the process as recommended by Fereday & Muir-Cochrane, (2006). A total of nine starting recommendations were generated for Round 1. These recommendations were presented to Youth Delphi Expert Panel Members.

**Table 2:** Open-ended questions used to formulate Delphi Round 1 starting recommendations

	Questions
1.	What do you think was done well during the COVID-19 pandemic in terms of the public health response? (i.e., public health response includes the restrictions that were put into place, the information provided to the public, etc.)
2.	What do you think should be done differently next time in terms of the public health response?
3.	How should government and policy makers be preparing now for the next pandemic or public health emergency?
4.	How should schools be preparing now for the next pandemic or public health emergency?
5.	How should employers be preparing now for the next pandemic or public health emergency?
6.	How should health service organizations be preparing now for the next pandemic or public health emergency?
7.	How should government and policy makers respond differently to the next pandemic or public health emergency?
8.	How should schools respond differently to the next pandemic or public health emergency?
9.	How should employers respond differently to the next pandemic or public health emergency?
10.	How should health service organizations respond differently to the next pandemic or public health emergency?
11.	Please specify: How should they respond differently to the next pandemic or public health emergency?
12.	Who should be involved in making decisions about public health responses to the next pandemic or public health emergency? How should they be involved?

### Delphi procedure

The study took place over three rounds, between July 2022 and April 2023. Participants were invited via email at the beginning of each round, with a link to the Delphi survey. Completion of each round required Panel Members to rate each recommendation item using a 7-point Likert scale (1 ‘one of the least important’ to 7 ‘one of the most important’), indicating the importance of the item. Panel Members were invited to provide comments and/or edits on the recommendation item using an open-ended response field in each round. In Rounds 1 and 2, participants were provided the opportunity to create their own recommendations. Demographic characteristics (age, gender identity, race/ethnicity, province/territory) were obtained in Round 1.

**Fig. 1:**
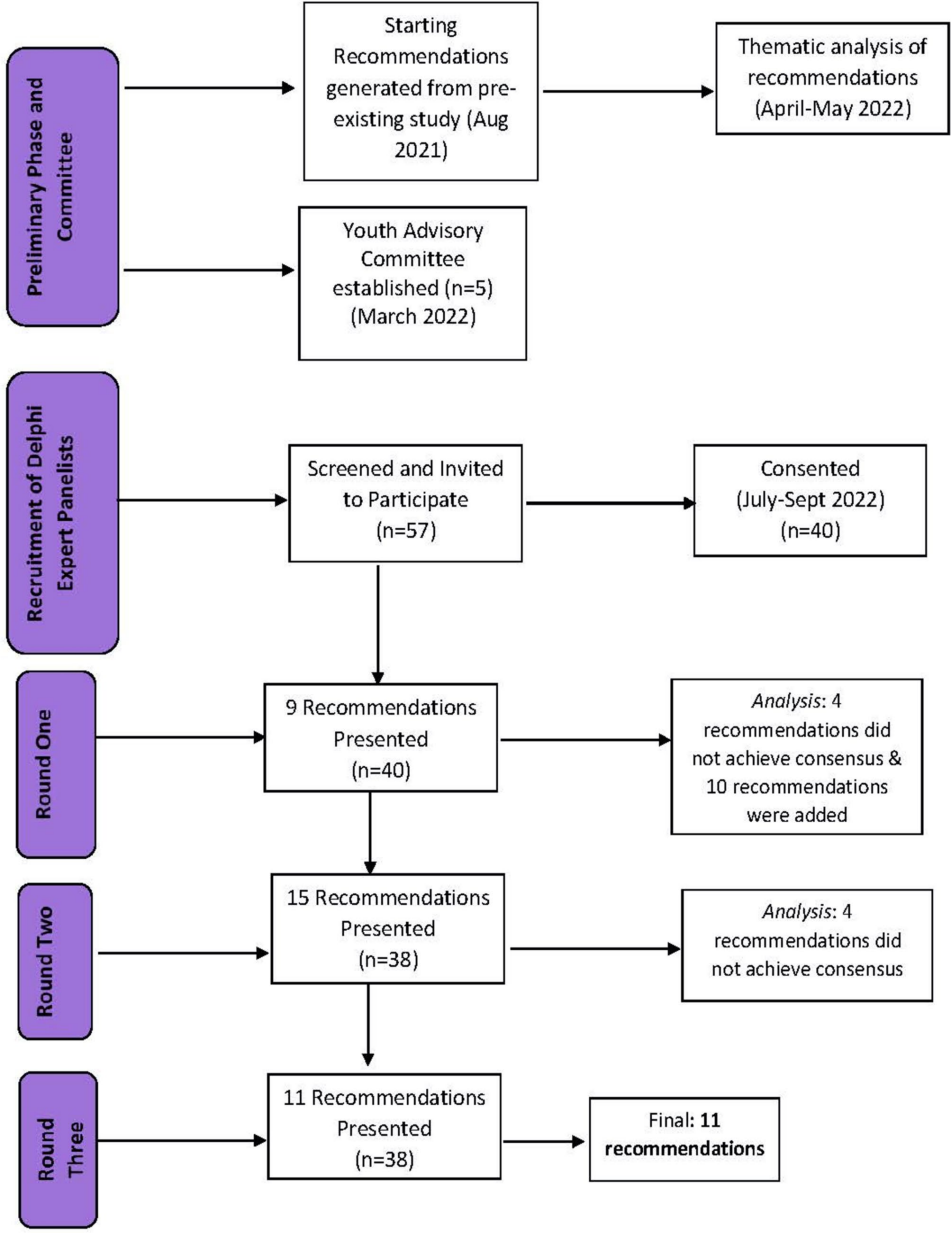
Flow diagram of Delphi rounds on planning for future pandemics or public health emergencies

Delphi rounds were kept open for 3–4 weeks, with completion reminder emails sent every week. De-identified quantitative and qualitative findings from Round 1 were included in Rounds 2 and 3 (Fig. 1). Results of each round were presented to the Youth Expert Advisory Committee to review the items that achieved consensus and the language of each item. Edits from the Panel Members and suggestions from the Youth Expert Advisory Committee were taken forward to the next round (see Table 3).

**Table 3:** Initial and final recommendation items ranked by Youth Delphi Expert Panel Members

	Initial recommendation	Final recommendation
B	Ensure that clear pandemic policies are in place	Ensure that pandemic policies are clear to the public
E	Provide information about a pandemic as clearly as possible	Provide information about a pandemic that is clear, easy to understand and accessible in written and spoken languages
F	Include scientists and health professionals in making decisions on pandemic policies (e.g., physicians, mental health practitioners, public health experts, and researchers)	Prioritize scientists and health professionals as the leads on decisions related to pandemic policies (e.g., physicians, mental health practitioners, public health experts, and researchers)
H	Ensure vaccines are made available and distributed efficiently	Ensure that vaccines are easy to access, distributed efficiently, and equitably available to individuals in different communities (prioritizing those at-risk, lower-income individuals, racialized populations, etc.)
J	Ensure governments and other decision-makers prepare in advance for a future pandemic and implement policies and practice plans that are supported by science	Ensure governments and other decision-makers prepare in advance for a future pandemic and implement policies and practice plans that are supported by science
K	Implement financial aid programs and ensure they are accessible for youth who need them	Implement financial aid programs and ensure they are accessible for youth who need them
L	Ensure mental health and substance use services are easily accessible, timely, and well-known to youth in their schools, workplaces, and communities	Ensure mental health and substance use services are easily accessible, timely, and well-known to youth in their schools, workplaces, and communities
M	Fund mental health and substance use services, hire more mental health professionals, and ensure services are free or inexpensive	Fund mental health and substance use services, hire more mental health professionals, and ensure services are free or inexpensive
N	Ensure schools, workplaces, and mental health and substance use services offer a variety in-person and virtual options (e.g., individual or group support) for youth to choose from	Ensure schools, workplaces, communities, and mental health and substance use services offer a variety of in-person and virtual options for youth to choose from
P	Ensure schools and workplaces continue to prioritize the health, safety, and well-being of their students and employees	Ensure schools and workplaces continue to prioritize the health, safety, and well-being of their students and employees
Q	Provide employees with paid sick days and mental health days	Provide employees with paid sick days, mental health days and holidays

### Data analysis

Statistical analyses were performed using Stata 16.1 (StataCorp LLC, College Station, TX). Demographic data were analysed using descriptive statistics. The percentage of participants who highly endorsed (i.e., ranking a 6 or 7 on a 7-point Likert scale) recommendations was calculated for importance in each round. Following previous literature (Hasson et al., 2000), a priori consensus was considered achieved if ≥ 70% of the entire group rated items at a 6 or 7 (on a 7-point Likert scale). If ≥ 70% of the entire group did not rate items at a 6 or 7, we considered consensus achieved if ≥ 70% of subgroups of youth (e.g., age, race/ethnicity, gender and sexual identities, and urban/rural location) rated items at a 6 or 7. Items that did not achieve consensus were dropped in subsequent rounds. In Rounds 2 and 3, Youth Panel Members were presented with their individual and group mean score of each recommendation item in the prior round and asked to re-rate the items. Content analysis (Keeney, 2011) was used for qualitative responses in Rounds 1 and 2. New recommendation items were created if statements and areas were relevant and recurring. The recommendations that achieved consensus in Round 3 were presented to the Youth Expert Advisory Committee in a final meeting to ensure cohesion and relevancy of the recommendations.

## Results

A total of *n* = 40 youth participated in Round 1. Participation rates between Round 1 and Round 2 were 95% (*n* = 38 youth) and from Round 2 to Round 3, 100% (*n* = 38 youth). Youth primarily identified as a girl/woman (cis, Trans) (50%), from Central Canada (Ontario, Quebec) (47.5%), and living in urban areas (65.0%) (Table 4).

**Table 4:** Select demographic characteristics of Youth Delphi Expert Panel Members, Round 1 (*n* = 40)

Category		Mean (range)
Age (years)		20.0 (14–25)
		***n*** (%)
Age category (years)	14–16	7 (17.5)
	17–21	17 (42.5)
	22–25	16 (40.0)
Gender identity	Boy/man (cis, trans)	12 (30.0)
	Girl/woman (cis, trans)	20 (50.0)
	Nonbinary and gender-diverse	8 (20.0)
Ethnicity	Indigenous (in Canada)	1 (2.6)
	Asian	11 (28.2)
	Black	1 (2.6)
	Middle Eastern	1 (2.6)
	Mixed race	8 (20.5)
	White	17 (43.6)
Sexual identity	Straight	19 (47.5)
	2SLGBQ+	21 (52.5)
Born in Canada	Yes	29 (72.5)
	No	11 (27.5)
Region^a^	Prairies	9 (22.5)
	Western Canada	9 (22.5)
	Eastern Canada	3 (7.5)
	Central Canada	19 (47.5)
Area of residence	Large city or suburbs	26 (65.0)
	Small city, town, rural	14 (35.0)

^a^Provinces represented in each region: Prairies, Alberta; Western, British Columbia; Eastern Canada, New Brunswick, Nova Scotia; Central Canada, Ontario, Quebec

### Round one

Youth Delphi Expert Panel Members in Round 1 ranked nine starting recommendations on level of importance (Table 5). Round 1 indicated that level of agreement was highest for Recommendations E (85.0%), F (85.0%), and H (82.5%). Nonbinary and gender diverse youth (75.0%) and those 14–16 years of age (71.4%) prioritized Recommendation B. Recommendation C was prioritized by youth 14–16 years of age (71.4%) (Table 5).

**Table 5:** Percentage of Delphi experts rating recommendations at 6 or 7, all rounds

Recommendations	Round 1 (*n* = 40) ***n*** **(%)**	Round 2 (*n* = 38) ***n* (%)**	Round 3 (*n* = 38) ***n* (%)**
A. Implement lockdowns and public health measures immediately	Did not achieve consensus		
B. Ensure that pandemic policies are clear to the public	6 (75.0%) Nonbinary and gender-diverse 5 (71.4%) 14–16 years	14 (70.0%) 2SLGBQ+ 12 (80.0%) 22–25 years 17 (80.9%) Indigenous, Asian, Black 15 (78.9%) girls/women 18 (72.0%) urban	28 (73.7%) all youth
C. Enforce public health measures strictly and consistently	5 (71.4%) 14–16 years	Did not achieve consensus	
D. Implement lockdowns for one long stretch instead of many short periods	Did not achieve consensus		
E. Provide information about a pandemic that is clear, easy to understand and accessible in written and spoken languages	34 (85.0%) all youth^a^	32 (86.5%) all youth^a^	33 (86.8%) all youth^a^
F. Prioritize scientists and health professionals as the leads on decisions related to pandemic policies (e.g., physicians, mental health practitioners, public health experts, and researchers)	34 (85.0%) all youth^a^	30 (81.1%) all youth^a^	29 (76.3%) all youth
G. Include the public in making decisions on pandemic policies	Did not achieve consensus		
H. Ensure that vaccines are easy to access, distributed efficiently and equitably available to individuals in different communities (e.g., prioritizing those at high risk, lower-income individuals, racialized populations, etc.)	33 (82.5%) all youth^a^	30 (81.1%) all youth^a^	28 (73.7%) all youth
I. Include youth in making decisions on pandemic policies	Did not achieve consensus		
J. Ensure governments and other decision-makers prepare in advance for a future pandemic and implement policies and practice plans that are supported by science		12 (70.6%) White youth 9 (75.0%) boys/men 13 (86.7%) 22–25 years	12 (70.6%) White 14 (73.7%) girls/women 14 (70.0%) 2SLGBQ+ 12 (80.0%) 22–25 years
K. Implement financial aid programs and ensure they are accessible for youth who need them		5 (71.4%) Nonbinary and gender-diverse	13 (76.5%) White 6 (85.7%) Nonbinary and gender diverse 13 (72.2%) Straight 5 (71.4%) 14–16 years
L. Ensure mental health and substance use services are easily accessible, timely and well-known to youth in their schools, workplaces, and communities		29 (75.3%) all youth	32 (84.2%) all youth^a^
M. Fund mental health and substance use services, hire more mental health professionals, and ensure services are free or inexpensive		29 (75.7%) all youth	31 (81.6%) all youth^a^
N. Ensure schools, workplaces, communities, and mental health and substance use services offer a variety of in-person and virtual options for youth to choose from		15 (78.9%) girls/women	16 (76.2%) Indigenous, Asian, Black 14 (73.7%) girls/women 18 (72.0%) urban 13 (72.2%) straight 6 (85.7%) 14–16 years 12 (75.0%) 17–21 years
O. Support vital learning by providing students with technology and academic supports (e.g., laptops, accessible Wi-Fi, homework help, etc.)		Did not achieve consensus	
P. Ensure schools and workplaces prioritize the health, safety and well-being of their students and employees		5 (71.4%) nonbinary and gender-diverse 5 (71.4%) 14–16 years	5 (71.4%) nonbinary and gender diverse 18(72%) urban 14 (70.0%) 2SLGBQ+ 6 (85.7%) 14–16 years
Q. Provide employees with paid sick days, mental health days and other days off as needed		12 (70.1%) White 14 (73.7%) girl/women 18 (72.0%) urban	29 (76.3%) all youth
R. Consider the impact of pandemic-related decisions on people from all age groups and communities and listen to their perspectives		Did not achieve consensus	
S. Ensure all youth are provided with the opportunity to voice their opinion on pandemic planning strategies through different platforms (e.g., surveys, online forums, advisory boards, discussion groups, etc.)		Did not achieve consensus	

^a^Top recommendations in each round based on percentage highly endorsed on importance

Four recommendations did not achieve consensus and were dropped (Recommendations A, D, G, and I). These recommendations focused on lockdowns and inclusion of communities in making pandemic-related policies.

Panel members provided open-ended responses to create new recommendations in Round 1. Similar statements were grouped into areas, including the following: (i) financial aid; (ii) virtual and in-person health and social services; (iii) investment in MHSU services; (iv) perspectives and voices in pandemic-related decisions and strategies; and (v) student and employee health and safety. Ten new recommendations were added (Recommendations J–S) based on open-ended responses during this round.

### Round two

Youth Delphi Expert Panel Members ranked 15 recommendations in Round 2 on importance (Table 5). Round 2 indicated that the level of agreement was highest for Recommendations E (86.5%), F (81.1%), and H (81.1%). Recommendation B was prioritized by Indigenous, Asian, and Black youth (80.9%); 22–25 year old (80.0%); girls/women (78.9%); those living in urban areas (72%), and 2SLGBQ+ youth (70.0%). Recommendation J was prioritized by 22–25 year old youth (86.7%); boys/men (75.0%); and White youth (70.6%). Recommendation K was prioritized by nonbinary and gender diverse youth (71.4%). Recommendation N was prioritized by youth who identify as girls/women (78.9%). Recommendation P was prioritized by nonbinary and gender diverse youth (71.4%) and those 14–16 years of age (71.4%). Recommendation Q was prioritized by girls/women (73.7%); those living in large cities (72.0%); and White youth (70.1%) (Table 5).

Four recommendations did not achieve consensus and were therefore dropped from the next round (Recommendations C, O, R, and S). These recommendations focused on enforcing public health measures; supporting virtual learning for students; and giving communities the opportunity to voice their perspectives on planning strategies and pandemic-related decisions. No new recommendations were added.

### Round three

A total of eleven recommendations were presented in Round 3, and eleven recommendations achieved consensus (Table 5). Recommendations E (86.8%), L (84.2%), and M (81.6%) had the highest percentage of agreement in Round 3. The percentage of agreement on recommendations F and H was 76.3% and 73.7%, respectively. Subgroups of youth rated specific recommendations as important: Recommendation J was prioritized by 22–25-year-old youth (80.0%), girls/women (73.7%), White youth (70.6%), and 2SLGBQ+ youth (70.0%). Recommendation K was prioritized by nonbinary and gender-diverse youth (85.7%), White youth (76.5%), youth who identify as straight (72.2%), and 14–16-year-old youth (71.4%). Recommendation N was prioritized by 14–16-year-old youth (85.7%), Indigenous, Black, and Asian youth (76.2%); 17–21-year-old youth (75.0%); girls/women (73.7%); youth who identify as straight (72.2%); and urban youth (72.0%). Recommendation P was prioritized by 14–16-year-old youth (85.7%); nonbinary and gender-diverse youth (71.4%); and 2SLGBQ+ youth (70.0%) (Table 5).

## Discussion

To our knowledge, this national study is the first to define youth-developed recommendations on planning for future pandemics and public health emergencies in Canada. Youth achieved consensus on eleven recommendations in support of preparedness for the next pandemic or public health emergency. These recommendations focused on the provision of easily accessible, clear, and understandable information about pandemics; the efficient and equitable distribution of vaccines; awareness of accessible MHSU services in schools, workplaces, and communities; investment in free or inexpensive MHSU services; pandemic-related policy decisions led by health professionals and scientists; clear pandemic policies available to the public; evidence-informed pandemic preparedness; implementation of financial aid programs; the provision of in-person and virtual MHSU service options to youth, families, and the community; prioritization of the health, safety, and well-being of students and workplaces; and the provision of paid sick- and mental health-days.

The recommendations generated in this study align with previously published pandemic preparedness responses. For example, the American College of Physicians (Serchen et al., 2023) supports similar recommendations, including an evidence-based comprehensive pandemic preparedness plan; clear communication on pandemic-related information; the promotion of physical and mental well-being among populations; universal access to paid sick leave and time off; and the efficient and equitable distribution of vaccines. Similar recommendations were drafted for the Quebec government to inform planning of the next pandemic (Alami et al., 2021). These recommendations included improving communication between the public and the government; strengthening the role of knowledge-based agencies in making decisions; supporting digital health strategies and telehealth; and establishing reliable health information systems that can be shared with the public.

Results of this study indicate that youth recommend clear and accessible information about the pandemic. In order to follow through on this recommendation, Canada needs to strengthen its public health system and handling of misinformation (Julie et al., 2021). Indeed, one of the most pressing public health challenges experienced over the pandemic period was the extensive amount of misinformation circulated online about the pandemic, public health measures and policies (Morganstein, 2022). This misinformation was particularly acute for youth (Casero-Ripollés, 2020), who may not have had the capacity to discern and filter reliable and accurate information from misinformation (Strasser et al., 2022). For example, a systematic review and secondary data analysis reported that the frequency and consumption of COVID-19-related news was adversely associated with youth mental health concerns (Strasser et al., 2022). To counter the spread of misinformation, clear, accurate, and transparent information about public health policies and measures must be shared and widely accessible. This information needs to be delivered early on through communication methods tailored to youth and involve diverse youth in the co-design of messaging. A recent systematic review on communication interventions to combat COVID-19 vaccine misinformation (Whitehead et al., 2023) reported that the most effective strategies included adding misinformation warnings; using humour to convey messages; and highlighting that the evidence was generated through scientific consensus. In addition, a systematic review highlighted that information be developed by reliable and credible sources and tailored to different communities’ lived experiences, needs, and concerns (Morganstein, 2022).

Youth recommend that health professionals and scientists inform and lead pandemic and public health emergency–related decisions. While all youth in this study agreed that science plays a critical role in public health preparedness, a qualitative study of COVID-19 policy advisors reported the challenges that scientific advisors experienced over the COVID-19 period (Vickery et al., 2022). These challenges included the inability to stay up-to-date on the evidence given the overwhelming, rapid generation of evolving and sometimes conflicting evidence; scientific uncertainty about different pandemic-related scenarios; the misinterpretation and misapplication of evidence; concerns about research integrity; and the lack of clarity on the integration of multi-sectoral evidence. At the same time, scientific advisors reported that they experienced a lack of transparency with governmental decision-makers on how pandemic-related decisions were made (Vickery et al., 2022). Further, a qualitative study and repeated-measures cohort study reported that scientific and technical jargon can be alienating for the public, pointing to a need for better knowledge translation (Kirkpatrick et al., 2017).

To overcome these challenges, youth also recommended that scientists and health professionals lead pandemic-related decisions. This recommendation could be achieved by establishing a diverse, multidisciplinary, and integrated group of scientific experts, in collaboration with a youth advisory group. To inform and support these decisions, establishing and promoting tools based on open science principles and responsible data sharing, guided by Ownership, Control, Access and Possession (OCAP®) principles (First Nations Information Governance Centre, 2014) and Engagement Governance, Access and Protection (EGAP) framework (Black Health Equity Working Group, 2021) is critical. Further, there is a need to establish a rigorous review system that ensures rapid access to clear and reliable evidence, as well as decision-support frameworks to support scientific integrity and transparency to communities (Vickery et al., 2022).

A recommendation endorsed among subgroups of youth (e.g., nonbinary and gender diverse, White, straight, and 14–16 years) identified a need for financial aid programs; specifically, the implementation of accessible financial aid programs in the context of public health emergencies and future pandemics. The COVID-19 pandemic period was associated with far-reaching negative economic consequences, including recession, unemployment, business closures, and by inflation and rising prices for consumers (Goniewicz et al., 2023). Two previous reviews (Goniewicz et al., 2023) suggested that focusing on social protection measures, including promoting social welfare, targeted measures for vulnerable populations, and establishing a minimum standard of living (e.g., food, shelter, clothing, finances) could help buffer the shock of the next pandemic or public health emergency.

We would like to acknowledge some of the strengths and limitations of this study. Strengths include the authentic engagement of youth; recommendations that reflect youth’s lived experiences and needs; robust participation rates in each round; and recommendations that can inform public health planning for future pandemics and public health emergencies. Limitations are as follows: Fifty percent of the study sample identified as a girl/woman. The recommendations generated may not be representative of the perspectives of boys/men and nonbinary or gender diverse youth. Participants resided mainly in Central Canada (Ontario and Quebec) and may not represent youth perspectives from other provinces and territories. It is important to obtain these perspectives. Further, the Canadian health system is decentralized at the provincial/territorial level; therefore, implementation of these recommendations will vary by province and territory. Further, although the study took place within the context of COVID-19 and planning for future pandemics, these recommendations have relevance to other unexpected emergencies requiring rapid response, such as climate-related disasters, other environmental or public health emergencies, and/or sudden economic collapse of certain sectors of the economy.

Recognizing that these recommendations reflect youth needs and priorities for the next pandemic or public health emergency, future work is needed to support the translation of these recommendations into policy action. The Youth Expert Advisory Committee in this study recommended that youth collaborate with scientists and health professionals to support decision-making. As part of a knowledge dissemination strategy, a prior literature review has highlighted the important role of training researchers about engaging policy-makers, learning about the policy-making process and how to convey evidence to policy-makers (Scott et al., 2019). Other strategies include the identification of knowledge brokers, or an individual who moves the knowledge from knowledge creators (e.g., youth and the research team) to knowledge users (e.g., policy- and decision-makers); face-to-face contact; and communication through various media platforms (Ward et al., 2009).

## Conclusions

These recommendations matter because they can be used to guide Canada’s preparedness and response strategies and policies for future pandemics and other public health or environmental emergencies, with the aim of providing optimal support to youth, families, and communities. The youth mental health consequences of the COVID pandemic were significant and it has provided us an opportunity to strengthen our public health system and responses to future pandemics and emergencies. Together, with health and other representatives at the federal, provincial, and local levels, youth and other community leaders should build an implementation plan for these recommendations, with a continued focus on equity, and secure funding to support their implementation, monitoring, and evaluation.

## Contributions to knowledge

What does this study add to existing knowledge?

- This national study is the first to define youth-developed recommendations on public health planning for future pandemics and emergencies in Canada.
- Youth endorse future pandemic and emergency strategies that include the following: clear, understandable pandemic information; efficient and effective distribution of vaccines; greater awareness and investment in timely and accessible mental health and substance use services; and health professionals and scientists leading pandemic-related decisions.

What are the key implications for public health interventions, practice, or policy?

- Strengthening Canada’s public health system and handling of misinformation is important. One way to address this is through wide dissemination of clear, accurate, and transparent information about public health policy measures that are tailored to diverse youth.
- A diverse, multidisciplinary, and integrated group of scientific experts and youth advisory group should be established to inform and lead public health emergency and pandemic-related decisions.

## Data Availability

The data used and/or analysed during the current study are available from the corresponding author on reasonable request.

## References

[CR1] Alami, H., Lehoux, P., Fleet, R., Fortin, J. P., Liu, J., Attieh, R., & Ag Ahmed, M. A. (2021). How can health systems better prepare for the next pandemic? Lessons learned from the management of COVID-19 in Quebec (Canada). *Frontiers In Public Health,**9*, Article 671833. 10.3389/fpubh.2021.671833 34222176 10.3389/fpubh.2021.671833 PMC8249772

[CR2] Black Health Equity Working Group. (2021). Engagement, governance, access, and protection (EGAP): A data governance framework for health data collected from black communities. Retrieved from https://blackhealthequity.ca

[CR3] Blanchet-Cohen, N., McMillan, Z., & Greenwood, M. (2011). Indigenous youth engagement in Canada’s health care. *Pimatisiwin: A Journal of Aboriginal and Indigenous Community Health,**9*, 87–111.

[CR4] Casero-Ripollés, A. (2020). Impact of Covid-19 on the media system. Communicative and democratic consequences of news consumption during the outbreak. *Casero-Ripollés, Andreu (2020).“Impact of Covid-19 on the media system. Communicative and democratic consequences of news consumption during the outbreak”. El profesional de la información, 29*(2), e290223.

[CR5] Cauberghe, V., Van Wesenbeeck, I., De Jans, S., Hudders, L., & Ponnet, K. (2021). How adolescents use social media to cope with feelings of loneliness and anxiety during COVID-19 lockdown. *Cyberpsychology, Behavior, and Social Networking,**24*(4), 250–257. 10.1089/cyber.2020.0478 33185488 10.1089/cyber.2020.0478

[CR6] Chacon, N. C., Walia, N., Allen, A., Sciancalepore, A., Tiong, J., Quick, R., Mada, S., Diaz, M. A., & Rodriguez, I. (2021). Substance use during COVID-19 pandemic: Impact on the underserved communities. *Discoveries (Covington, La.),**9*(4), e141. 10.15190/d.2021.20 10.15190/d.2021.20 PMC8896880 35261922

[CR7] CIHR (2023). Strategy for patient-oriented research. Ottawa, ON: Canadian institutes of health research. Retrieved from https://cihr-irsc.gc.ca/e/48413.html

[CR8] Darnay, K., Hawke, L.D. Chaim, G., Henderson, J., & the INNOVATE Research Team. (2019). *INNOVATE research: Youth engagement guidebook for researchers*. Toronto, ON: Centre for addiction and mental health.

[CR9] Dewa, L. H., Crandell, C., Choong, E., Jaques, J., Bottle, A., Kilkenny, C., Lawrence-Jones, A., Di Simplicio, M., Nicholls, D., & Aylin, P. (2021). Ccopey: A mixed-methods coproduced study on the mental health status and coping strategies of young people during COVID-19 UK lockdown. *Journal of Adolescent Health,**68*(4), 666–675. 10.1016/j.jadohealth.2021.01.009 10.1016/j.jadohealth.2021.01.009 PMC9188746 33589305

[CR10] Duan, L., Shao, X., Wang, Y., Huang, Y., Miao, J., Yang, X., & Zhu, G. (2020). An investigation of mental health status of children and adolescents in China during the outbreak of COVID-19. *Journal Of Affective Disorders,**275*, 112–118. 10.1016/j.jad.2020.06.029 32658812 10.1016/j.jad.2020.06.029 PMC7329661

[CR11] Fante-Coleman, T., & Jackson-Best, F. (2020). Barriers and facilitators to accessing mental healthcare in Canada for Black youth: A scoping review. *Adolescent Research Review,**5*(2), 115–136. 10.1007/s40894-020-00133-2

[CR12] Fereday, J., & Muir-Cochrane, E. (2006). Demonstrating rigor using thematic analysis: A hybrid approach of inductive and deductive coding and theme development. *International Journal of Qualitative Methods,**5*(1), 80–92. 10.1177/160940690600500107

[CR13] Gómez, C. A., Kleinman, D. V., Pronk, N., Wrenn Gordon, G. L., Ochiai, E., Blakey, C., Johnson, A., & Brewer, K. H. (2021). Addressing health equity and social determinants of health through Healthy People 2030. *Journal of Public Health Management and Practice*. 10.1097/PHH.0000000000001297 33729197 10.1097/PHH.0000000000001297 PMC8478299

[CR14] Goniewicz, K., Khorram-Manesh, A., Burkle, F. M., Hertelendy, A. J., & Goniewicz, M. (2023). The European Union’s post-pandemic strategies for public health, economic recovery, and social resilience. *Global Transitions,**5*, 201–209. 10.1016/j.glt.2023.10.003

[CR15] Harris, P. A., Taylor, R., Thielke, R., Payne, J., Gonzalez, N., & Conde, J. G. (2009). Research electronic data capture (REDCap)–a metadata-driven methodology and workflow process for providing translational research informatics support. *Journal of Biomedical Informatics,**42*(2), 377–381. 10.1016/j.jbi.2008.08.010 18929686 10.1016/j.jbi.2008.08.010 PMC2700030

[CR16] Hasson, F., Keeney, S., & McKenna, H. (2000). Research guidelines for the delphi survey technique. *Journal of Advanced Nursing,**32*(4), 1008–1015. 11095242

[CR17] Hawke, L. D., Barbic, S. P., Voineskos, A., Szatmari, P., Cleverley, K., Hayes, E., Relihan, J., Daley, M., Courtney, D., Cheung, A., Darnay, K., & Henderson, J. L. (2020). Impacts of COVID-19 on Youth Mental Health, Substance Use, and Well-being: A Rapid Survey of Clinical and Community Samples: Répercussions de la COVID-19 sur la santé mentale, l’utilisation de substances et le bien-être des adolescents: Un sondage rapide d’échantillons cliniques et communautaires. *The Canadian Journal of Psychiatry,**65*(10), 701–709. 10.1177/0706743720940562 32662303 10.1177/0706743720940562 PMC7502874

[CR18] Hawke, L. D., Monga, S., Korczak, D., Hayes, E., Relihan, J., Darnay, K., Cleverley, Kristin, Lunsky, Yona, Szatmari, Peter, & Henderson, J. (2021a). Impacts of the COVID-19 pandemic on youth mental health among youth with physical health challenges. *Early Intervention in Psychiatry,**15*(5), 1146–1153. 10.1111/eip.13052 33047495 10.1111/eip.13052 PMC7675347

[CR19] Hawke, L. D., Szatmari, P., Cleverley, K., Courtney, D., Cheung, A., Voineskos, A. N., & Henderson, J. (2021). Youth in a pandemic: A longitudinal examination of youth mental health and substance use concerns during COVID-19. *BMJ Open,**11*(10), Article e049209. 10.1136/bmjopen-2021-049209 34716160 10.1136/bmjopen-2021-049209 PMC8561825

[CR20] Henderson, J. L., Hawke, L. D., & Relihan, J. (2018). Youth engagement in the YouthCan IMPACT trial. *Canadian Medical Association Journal,**190*(Suppl), S10. 10.1503/cmaj.180328 30404840 10.1503/cmaj.180328 PMC6472455

[CR21] Hsu, C. C., & Sandford, B. (2007). "The Delphi technique: Making sense of consensus". *Practical Assessment, Research and Evaluation, 12*(1), 10.

[CR22] Julie, P., Maria, O., Omolara, S., Brittany, M., Rachel, L., Sana, A., & Vivian, A. W. (2021). Public health measures to reduce the risk of SARS-CoV-2 transmission in Canada during the early days of the COVID-19 pandemic: A scoping review. *BMJ Open,**11*(3), Article e046177. 10.1136/bmjopen-2020-046177 10.1136/bmjopen-2020-046177 PMC7944419 33687956

[CR23] Jünger, S., Payne, S. A., Brine, J., Radbruch, L., & Brearley, S. G. (2017). Guidance on conducting and reporting delphi studies (CREDES) in palliative care: Recommendations based on a methodological systematic review. *Palliative Medicine,**31*(8), 684–706. 10.1177/0269216317690685 28190381 10.1177/0269216317690685

[CR24] Keeney, S. (2011). Conducting the research using the Delphi technique. In *The Delphi Technique in Nursing and Health Research* (pp. 69–83)

[CR25] Kidd, J. D., Paschen-Wolff, M. M., Mericle, A. A., Caceres, B. A., Drabble, L. A., & Hughes, T. L. (2022). A scoping review of alcohol, tobacco, and other drug use treatment interventions for sexual and gender minority populations. *Journal of Substance Abuse Treatment,**133*, Article 108539. 10.1016/j.jsat.2021.108539 34175174 10.1016/j.jsat.2021.108539 PMC8674383

[CR26] Kirkpatrick, E., Gaisford, W., Williams, E., Brindley, E., Tembo, D., & Wright, D. (2017). Understanding plain English summaries. A comparison of two approaches to improve the quality of plain English summaries in research reports. *Research Involvement and Engagement,**3*(1), Article 17. 10.1186/s40900-017-0064-0 29062542 10.1186/s40900-017-0064-0 PMC5632836

[CR27] Morganstein, J. C. (2022). Preparing for the next pandemic to protect public mental health: What have we learned from COVID-19? *Psychiatric Clinics of North America,**45*(1), 191–210. 10.1016/j.psc.2021.11.012 35219438 10.1016/j.psc.2021.11.012 PMC8585601

[CR28] Naff, D., Williams, S., Furman-Darby, J., & Yeung, M. (2022). The mental health impacts of COVID-19 on PK–12 students: A systematic review of emerging literature. *AERA Open,**8*, Article 23328584221084722. 10.1177/23328584221084722

[CR29] OCAP® is a registered trademark of the First Nations Information Governance Centre (FNIGC). (2014). Retrieved February 8 2025 from https://fnigc.ca/ocap-training/

[CR30] Powell, C. (2003). The delphi technique: Myths and realities. *Journal of Advanced Nursing,**41*(4), 376–382. 10.1046/j.1365-2648.2003.02537.x 12581103 10.1046/j.1365-2648.2003.02537.x

[CR31] Quinlan-Davidson, M., Cleverley, K., Barbic, S., Courtney, D., Dimitropoulos, G., Hawke, L. D., Nandlall, N., Ma, C., Prebeg, M., & Henderson, J. L. (2025). Are we out of the woods yet? Youth-developed recommendations on recovery from the COVID-19 pandemic: A national Delphi study. Can J Public Health. Epub ahead of print. 10.17269/s41997-025-01020-w 10.17269/s41997-025-01020-w PMC12753593 40240740

[CR32] Quinlan-Davidson, M., Shan, D., Courtney, D., Barbic, S., Cleverley, K., Hawke, L. D., Ma, C., Prebeg, M., Relihan, J., Szatmari, P., & Henderson, J. L. (2023). Associations over the COVID-19 pandemic period and the mental health and substance use of youth not in employment, education or training in Ontario, Canada: A longitudinal, cohort study. *Child and Adolescent Psychiatry and Mental Health,**17*(1), 105. 10.1186/s13034-023-00653-4 37679811 10.1186/s13034-023-00653-4 PMC10486040

[CR33] Roberts, T. K., & Fantz, C. R. (2014). Barriers to quality health care for the transgender population. *Clinical Biochemistry,**47*(10), 983–987. 10.1016/j.clinbiochem.2014.02.009 24560655 10.1016/j.clinbiochem.2014.02.009

[CR34] Schoon, I., Shukla, S., Verma, S., Terol, E., & Da Cunha, J. M. (2025). The COVID-19 pandemic and young people’s civic engagement: A scoping review. *Journal of Research on Adolescence,**35*(1), Article e13039. 10.1111/jora.13039 39616498 10.1111/jora.13039 PMC11758488

[CR35] Scott, J. T., Buckingham, L. J., Maton, S. L., Maton, K. I., & Crowley, D. M. (2019). Bridging the research-policy divide: Pathways to engagement and skill development. *American Journal of Orthopsychiatry,**89*(4), 434–441. 31305112 10.1037/ort0000389 PMC8547491

[CR36] Serchen, J., Cline, K., Mathew, S., & Hilden, D. (2023). Preparing for future pandemics and public health emergencies: An American College of Physicians policy position paper. *Annals of Internal Medicine,**176*(9), 1240–1244. 10.7326/m23-0768 37487216 10.7326/M23-0768

[CR37] Strasser, M. A., Sumner, P. J., & Meyer, D. (2022). COVID-19 news consumption and distress in young people: A systematic review. *Journal of Affective Disorders,**300*, 481–491. 10.1016/j.jad.2022.01.007 34990630 10.1016/j.jad.2022.01.007 PMC8742131

[CR38] Thomas, R. F., Marine, B., & Amanda, M. (2021). The world must prepare now for the next pandemic. *BMJ Global Health,**6*(3), e005184. 10.1136/bmjgh-2021-005184 10.1136/bmjgh-2021-005184 PMC7970315 33727280

[CR39] Vickery, J., Atkinson, P., Lin, L., Rubin, O., Upshur, R., Yeoh, E. K., Boyer, C., & Errett, N. A. (2022). Challenges to evidence-informed decision-making in the context of pandemics: Qualitative study of COVID-19 policy advisor perspectives. *BMJ Global Health*. 10.1136/bmjgh-2021-008268 35450862 10.1136/bmjgh-2021-008268 PMC9023846

[CR40] Ward, V., House, A., & Hamer, S. (2009). Knowledge brokering: The missing link in the evidence to action chain? *Evidence & Policy,**5*(3), 267–279. 10.1332/174426409x463811 21258626 10.1332/174426409X463811 PMC3024540

[CR41] Whitehead, H. S., French, C. E., Caldwell, D. M., Letley, L., & Mounier-Jack, S. (2023). A systematic review of communication interventions for countering vaccine misinformation. *Vaccine,**41*(5), 1018–1034. 10.1016/j.vaccine.2022.12.059 36628653 10.1016/j.vaccine.2022.12.059 PMC9829031

[CR42] WHO. (2025). *World report on social determinants of health equity*. Retrieved from Geneva

[CR43] Wilf, S., Wray-Lake, L., & Saavedra, J. A. (2023). Youth civic development amid the pandemic. *Current Opinion in Psychology,**52*, Article 101627. 10.1016/j.copsyc.2023.101627 37392503 10.1016/j.copsyc.2023.101627 PMC10266065

[CR44] Zhang, L., Zhang, D., Fang, J., Wan, Y., Tao, F., & Sun, Y. (2020). Assessment of mental health of Chinese primary school students before and after school closing and opening during the COVID-19 pandemic. *JAMA Network Open,**3*(9), Article e2021482. 10.1001/jamanetworkopen.2020.21482 32915233 10.1001/jamanetworkopen.2020.21482 PMC7489803

